# Factor Xa inhibitors versus vitamin K antagonist in morbidly obese patients with venous thromboembolism: a systematic review and meta-analysis

**DOI:** 10.1186/s12872-023-03067-4

**Published:** 2023-02-22

**Authors:** Dae Yong Park, Seokyung An, Abdul Wahab Arif, Muhammad Khawar Sana, Aviral Vij

**Affiliations:** 1 Department of Medicine, John H. Stroger Jr Hospital of Cook County, Chicago, IL USA; 2 Department of Biomedical Science, Seoul National University Graduate School, Seoul, Korea; 3 Division of Cardiology, Cook County Health, Chicago, IL USA; 4 Division of Cardiology, Rush Medical College, Chicago, IL USA

**Keywords:** Oral anticoagulant, Xabans, Venous thromboembolism, Obesity

## Abstract

**Introduction:**

Guidelines have endorsed non-vitamin K antagonist oral anticoagulants (NOACs), consisting of factor Xa inhibitors (xabans) and direct thrombin inhibitors, as the first line of treatment in venous thromboembolism (VTE) and atrial fibrillation. However, morbidly obese patients were under-represented in landmark trials of NOACs. Therefore, this study aimed to systematically review and perform a meta-analysis of studies on xabans versus vitamin K antagonist (VKA) in this high-risk population with VTE.

**Methods:**

PubMed, Embase, Medline, Cochrane library, and Google Scholar databases were searched to identify studies that compared xabans and VKA in treating morbidly obese patients with VTE. Morbid obesity was defined as body weight ≥ 120 kg or BMI ≥ 40 kg/m^2^. Outcomes of interest included recurrent VTE, major bleeding, and clinically relevant non-major bleeding (CRNMB).

**Results:**

Eight studies comprising 30,895 patients were included. A total of 12,755 patients received xabans while 18,140 received VKAs. No significant difference in the odds of recurrent VTE (OR 0.75, 95% CI 0.55–1.01) and CRNMB (OR 0.69, 95% CI 0.44–1.09) was observed between the xabans group and the VKA group. However, the xabans group was associated with lower odds of major bleeding (OR 0.70, 95% CI 0.59–0.83).

**Conclusion:**

Xabans have lower odds of major bleeding but similar odds of recurrent VTE when compared with VKAs in treating VTE in morbidly obese patients. Large registry analyses or future randomized controlled trials will be helpful in confirming these findings.

**Supplementary Information:**

The online version contains supplementary material available at 10.1186/s12872-023-03067-4.

## Introduction

Non-vitamin K antagonist oral anticoagulants (NOACs) have revolutionized the anticoagulation therapies which were largely reliant on heparin derived agents and warfarin until 2009 [1]. These newer agents have fewer dietary and drug interactions, less fatal bleeding, and do not need significant monitoring of drug levels [2]. With consistent results across the population in large scale studies, NOACs are recommended as the agent of choice in prominent clinical guidelines in treating venous thromboembolism (VTE) and atrial fibrillation (AF) [3, 4].

The safety and efficacy of NOACs in obesity is poorly understood. Reduced peak levels due to underdosing, shorter half-lives, and increased volume of distribution in those with higher body weight are few of the concerns raised with use of NOACs in obese patients. The prevalence of obesity in the United States was 42.4% (2017–2018) and is predicted to increase to nearly 50% by 2030, with almost one in four adults at risk of severe obesity [5, 6]. With limited representation of obese patients in published studies, International Society on Thrombosis and Hemostasis (ISTH) did not previously recommend the use of NOACs in patients with body mass index (BMI) > 40 kg/m^2^ or weight > 120 kg [7]. While the phase III trials of NOACs included subgroup analysis of obese patients, their sample sizes were relatively small and mostly limited to BMI and weight cutoff at > 35 kg/m^2^ and > 100 kg, respectively [7]. However, with emerging data from largely observational studies, ISTH provided a guideline update suggesting use of standard dose rivaroxaban and apixaban for VTE treatment regardless of obesity [8].

NOACs typically refer to four drugs: one oral direct thrombin inhibitor—dabigatran, and three oral factor Xa inhibitors (xabans)—rivaroxaban, apixaban, and edoxaban [9]. Xabans are favored more over direct thrombin inhibitors due better availability, once-daily dosing (rivaroxaban), approval in chronic kidney disease or end-stage renal disease (apixaban), and cost [8, 10–12]. The objective of this study is to perform a systematic review and updated meta-analysis examining the effect and safety of xabans in comparison to the traditionally used vitamin K antagonist (VKA) in treating VTE in patients with high BMI or weight.

## Methods

### Search strategy and inclusion criteria

This systematic review and meta-analysis adhered to the Preferred Reporting Items for Systematic Reviews and Meta-Analyses (PRISMA) guideline [13]. Two independent authors (D.P. and S.A.) searched relevant literatures in PubMed, Embase, Medline, Cochrane library, and Google Scholar published up to January 31, 2022. The following keywords were used for disease: “venous thromboembolism”, “morbid obesity”, “morbidly obese”, “obesity”, and “obese”. To define exposure, the following keywords were used: “oral anticoagulants”, “xabans”, “rivaroxaban”, “apixaban”, “edoxaban”, “vitamin K antagonist”, and “warfarin”. Studies written in English were reviewed to select eligible studies on xabans versus VKA on VTE recurrence, major bleeding, and CRNMB. Additional studies were also manually searched through the references cited in reviews. Randomized clinical trials and cohort studies were included as original articles. For studies with duplicative population, the study with more comprehensive data was selected. The following studies were excluded: studies that did not report outcome in morbidly obese patients, studies that included dabigatran in the case group, studies that did not have those taking VKA in the control group, and review articles. The study protocol has been published online: 10.17605/OSF.IO/9NK63.

### Definition of outcomes

In this study, morbid obesity was defined as BMI ≥ 40 kg/m^2^ or body weight ≥ 120 kg. The cut-off of 120 kg in defining morbid obesity was used in accordance with the ISTH guidelines and many previous studies [8, 14–16]. VTE recurrence was defined as new or worsening deep vein thrombosis or pulmonary embolism proven by imaging studies or clinical judgment. Occurrences of bleeding were classified using the criteria set by ISTH. Major bleeding was defined as symptomatic manifestation with fatal bleeding, bleeding in a critical area or organ, hemoglobin drop ≥ 2 g/dL, or blood transfusion ≥ 2 units [17]. Clinically relevant non-major bleeding (CRNMB) was defined by bleeding requiring medical encounter, care, or intervention, but not meeting the criteria for major bleeding [17].

### Assessment of bias

Funnel plot showing the scatter plot of the odds ratio against the standard error in a logarithmic scale was performed to evaluate for publication bias, after which Begg-Mazumdar and Egger tests were applied whose *P*-value < 0.05 indicates statistically significant publication bias. Other potential biases in the selected studies were evaluated using the Newcastle–Ottawa scale, which awards points to 9 different items distributed among categories of case–control selection, comparability, and outcome, and signifies less risk of bias with higher overall scores [18]. Quality of evidence in the integrated outcomes were assessed using the Grading of Recommendations, Assessment, Development and Evaluations (GRADE) criteria [19].

### Statistical analysis

Integrated odds ratio (OR) and 95% confidence intervals (95% CI) were generated by applying random effects model based on the DerSimonian-Laird method. The consistent use of random effects model was preplanned given the relatively fewer number of studies and the expected heterogeneity among the trials. To assess the heterogeneity of the studies, Higgins and Thompson’s I2 statistics were calculated. The I2 measure ranges from 0 to 100%, which suggests that less than 50%, 50–74%, and over 75% is considered as low, moderate, and substantial heterogeneity, respectively. Two sensitivity analyses were performed: one excluding studies with high risk of bias, and another excluding studies that used body weight ≥ 120 kg instead of BMI ≥ 40 kg/m^2^ to define morbid obesity. All statistical analyses were conducted using the *meta* package in R version 4.0.2.

## Results

### Included studies

**Fig. 1:**
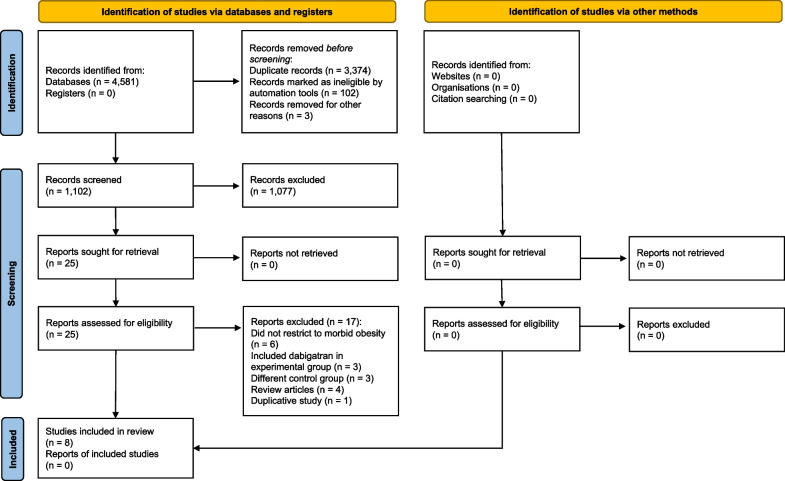
PRISMA flow diagram for studies comparing factor Xa inhibitors and vitamin K antagonist. This figure illustrates the search process conducted to identify studies meeting the inclusion criteria in accordance with PRISMA guidelines

Initial search of literature identified 1,102 studies, of which 1,094 were excluded after examining the title, abstract, and full text (Fig. 1). Eight studies were selected in the final analysis: 1 post hoc analysis [14] and 7 retrospective cohort studies [15, 20–25]. Two studies originally [20, 21] included patients with BMI > 30 kg/m^2^, so data from their subgroup analyses of patients with BMI > 40 kg/m^2^ were used.

### Risk of bias

Funnel plot analysis of the included studies showed no evidence of publication bias on the effect of xabans compared to VKA (Additional file 1: Figure S1). Other potential biases were evaluated to be low to moderate primarily because of inadequacy of outcomes and compromised comparability (Additional file 1: Table S1). Sa et al. [24] and Cohen et al. [20] had the highest risk of bias. These biases, in addition to moderate imprecision due to paucity of included studies, moderate inconsistencies due to heterogeneities, and moderate indirectness due to suboptimal comparability, led to moderate, instead of high, GRADE ratings (Additional file 1: Table S2). PRISMA checklist can be found in Additional file 1: Table S3.

### Characteristics

Main characteristics and demographics of the studies are summarized in Tables 1 and 2. Inclusion and exclusion criteria applied in the studies are shown in Additional file 1: Table S4. A total of 30,895 patients were studied, with 12,755 patients (41.3%) receiving xabans and 18,140 patients (58.7%) receiving VKAs. Three studies [15, 21, 25] solely included patients who received rivaroxaban in the case group, while 3 studies [14, 20, 22] included only those who received apixaban. The remaining 2 studies [23, 24] included different proportions of rivaroxaban, apixaban, and edoxaban.

**Table 1:** Main characteristics of selected studies in the meta-analysis

Author	Years	Type of study	Country	Follow up (days)	Total	Case^a^	Control^b^	Proportion of Xabans
Kushnir et al	2019	Retrospective	US	196	366	199	167	R (76.4%), A (23.6%)
Sa et al	2019	Retrospective	Canada	365	133	71	62	R (80%), A and E (20%)
Spyropoulos et al	2019	Retrospective	US	300	5789	2890	2890	R (100%)
Perales et al	2020	Retrospective	US	365	109	47	62	R (100%)
Cohen et al	2021	Retrospective	US	180	19,751	7411	12,340	A (100%)
Cohen et al	2021	Post-hoc analysis	Multinational	180	263	126	137	A (100%)
Costa et al	2021	Retrospective	US	365	3394	1697	1697	R (100%)
Crouch et al	2021	Retrospective	US	365	1099	314	785	A (100%)

*A* apixaban, *E* edoxaban, *R* rivaroxaban, *US* United States, *xabans* factor Xa inhibitors

^a^Number of patients who received factor Xa inhibitors

^b^Number of patients who received warfarin

**Table 2:** Demographics of the studies

Author	Case age (years, mean ± SD)	Control age (years, mean ± SD)	Case^a^ (Male %)	Control^b^ (Male %)	Case BMI (kg/m^2^, mean ± SD)	Control BMI (kg/m^2^, mean ± SD)	Case PE (%)	Control PE (%)	Case provoked VTE (%)	Control provoked VTE (%)
Kushnir et al	52.6 ± 14.5	58.1 ± 15.1	32	29	43.7 ± NR	45.3 ± NR	NR	NR	NR	NR
Sa et al	NR	NR	NR	NR	NR	NR	NR	NR	NR	NR
Spyropoulous et al	53.3 ± 12.9	53.1 ± 13.1	39.5	39.8	NR	NR	NR	NR	NR	NR
Perales et al	56 ± 14	55 ± 15	52	55	45 ± NR	44 ± NR	31	41	NR	NR
Cohen et al.^a^	62.3 ± 13.9	62.1 ± 14.0	36.5	36.6	NR	NR	57.0	57.1	65.8	65.7
Cohen et al.^b^	53.7 ± 13.4	53.2 ± 13.2	37.3	32.1	45.2 ± 5.5	45.3 ± 5.5	NR	NR	5.6	13.0
Costa et al	NR	NR	NR	NR	NR	NR	NR	NR	NR	NR
Crouch et al	59.3 ± 13.9	57.7 ± 14.0	52.2	43.9	44.1 ± 6.8	47.1 ± 10.4	NR	NR	NR	NR

*SD* standard deviation, *BMI* body mass index, *kg/m*^*2*^ kilograms per meter squared, *PE* pulmonary embolism, *VTE* venous thromboembolism, *NR* not reported

^a^Patients who received factor Xa inhibitors

^b^Patients who received warfarin

^c^Retrospective cohort study

^d^Post-hoc analysis

### Outcomes

**Fig. 2:**
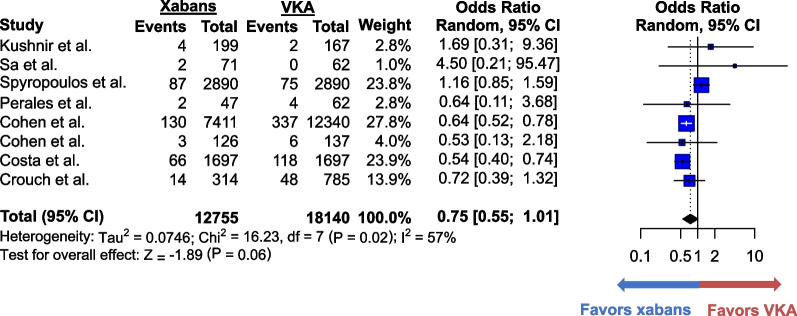
Forest plots for the comparative analysis of the odds of recurrent venous thromboembolism in all the selected studies. Forest plots show the odds ratio (blue box) of recurrent venous thromboembolism reported in each study. The size of the blue boxes corresponds to the weight given to the study. Vertical lines represent 95% confidence interval (CI) of each odds ratio. Pooled odds ratio is shown at the bottom bolded line and is represented by a black diamond. Odds ratio over 1 favors vitamin K antagonists whereas that below 1 favors xabans. Abbreviations: CI = confidence interval; VKA = vitamin K antagonist; xaban = factor Xa inhibitor

**Fig. 3:**
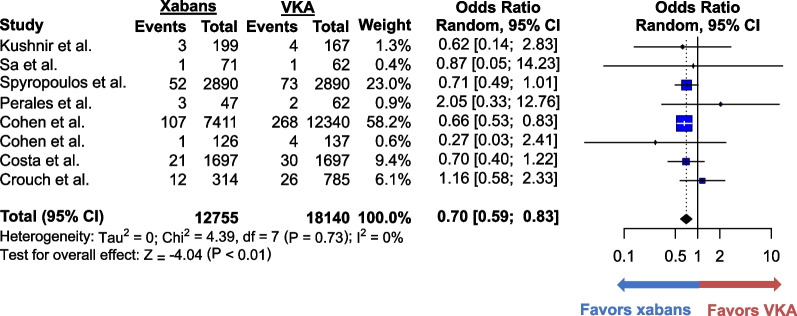
Forest plots for the comparative analysis of the odds of major bleeding in all the selected studies. Forest plots show the odds ratio (blue box) of major bleeding reported in each study. The size of the blue boxes corresponds to the weight given to the study. Vertical lines represent 95% confidence interval (CI) of each odds ratio. Pooled odds ratio is shown at the bottom bolded line and is represented by a black diamond. Odds ratio over 1 favors vitamin K antagonists whereas that below 1 favors xabans. Abbreviations: CI = confidence interval; VKA = vitamin K antagonist; xaban = factor Xa inhibitor

**Fig. 4:**
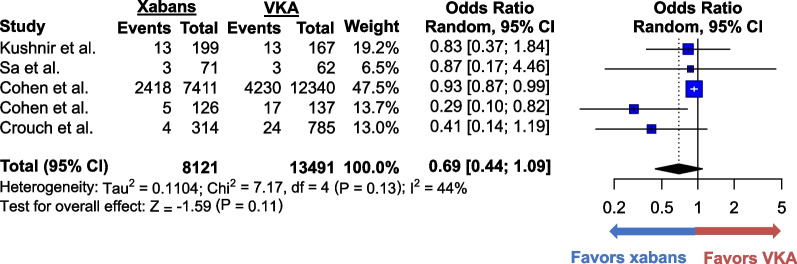
Forest plots for the comparative analysis of the odds of clinically relevant non-major bleeding in selected studies. Forest plots show the odds ratio (blue box) of clinically relevant non-major bleeding reported in each study. The size of the blue boxes corresponds to the weight given to the study. Vertical lines represent 95% confidence interval of each odds ratio. Pooled odds ratio is shown at the bottom bolded line and is represented by a black diamond. Odds ratio over 1 favors vitamin K antagonists whereas that below 1 favors xabans. Abbreviations: CI = confidence interval; VKA = vitamin K antagonist; xaban = factor Xa inhibitor

VTE recurred in 2.4% (308 of 12,755) of patients who took xabans and in 3.1% (590 of 18,140) of those who took VKAs. The difference in VTE recurrences between the 2 groups was not statistically significant (OR 0.75; 95% CI 0.55–1.01, *p* = 0.06) (Fig. 2). The heterogeneity was moderate (I^2^ = 57%), and the quality of evidence was moderate (Additional file 1: Table S2). Sensitivity analysis excluding studies with high risk of bias yielded similar results (Additional file 1: Figure S2). Additional sensitivity analysis excluding studies that defined morbid obesity as body weight ≥ 120 kg instead of BMI ≥ 40 kg/m^2^ also produced similar results (Additional file 1: Figure S3). Major bleeding occurred in 1.6% (200 of 12,755) of the xaban group and in 2.2% (408 of 18,140) of the VKA group. The odds of major bleeding were lower in the former group (OR 0.70; 95% CI 0.59–0.83, *p* < 0.01) (Fig. 3). The heterogeneity was minimal (I^2^ = 0%), and the quality of evidence was moderate. Only four studies reported CRNMB, which occurred in 30.1% (2,444 of 8,121) of patients who received xabans and in 31.8% (4,288 of 13,491) of patients who received VKAs (Fig. 4). No statistically significant difference was found between the two groups (OR 0.69; 95% CI 0.44–1.09, *p* = 0.11). The heterogeneity was low (I^2^ = 44%), and the quality of evidence for CRNMB was also moderate.

## Discussion

We investigated the effectiveness of xabans versus VKAs in morbidly obese patients with VTE. Our results show no statistical difference in recurrent VTE or CRNMB between xabans and VKA, and lower odds of major bleeding in the xabans group. Therefore, our findings suggest that xabans can be used to treat morbidly obese patients with VTE who were previously placed on VKAs that inconveniently required bridging, frequent blood draws for monitoring, and readmissions for sub- and supratherapeutic prothrombin time. While there have been concerns of low efficacy of xabans in obese patients due to fixed-dosage regimen, recent data has been reassuring [26–28]. To date, our review is the largest and most up-to-date study on the subject and support the growing evidence of safety and efficacy of xabans in treating morbidly obese patients with VTE.

Comparative analysis of the odds of recurrent VTE yielded substantial heterogeneity with Higgins and Thompson’s I2 statistic of 63%. This can be explained by several factors that include differences in the sample size and varying inclusion and exclusion criteria among the studies (Table 2). For instance, Sa et al. only included a total of 133 patients in both arms with no recurrence of VTE observed in the warfarin group, which generated an outlying odds ratio. On the other hand, Sa et al. [24], Cohen et al. [14, 20], and Costa et al. [21] excluded patients with cancers while others reported no restrictions. Xaban of choice also varied among the studies (Table 1).

Our results are consistent with those of previous studies, including sub-analyses of Einstein-DVT, Einstein-PE, and XALIA trials which found that fixed-dose rivaroxaban had a comparable efficacy and safety profile as that of heparin products or VKAs in patients with high BMI [29, 30]. In 2020, Elshafei et al. performed a meta-analysis evaluating VTE recurrence and major bleeding in 6,575 patients with BMI > 40 kg/m^2^ treated with either DOACs or warfarin, with results showing non-inferiority of DOACs with regards to VTE recurrence (OR 1.07, 95% CI 0.93–1.23) and no difference in the odds of major bleeding (OR 0.80, 95% CI 0.54–1.17) [31]. However, they could not rule out publication bias given asymmetry of the funnel plot. Moreover, analysis of VTE recurrence excluded one study that had reported VTE recurrence in rates, and their safety outcome assessing major bleeding excluded one study that had reported only a composite of major bleeding events and CRNMB.

Despite obesity being a risk factor for recurrent VTE, the overall incidence of recurrent VTE in our study was similar to that of prior studies [29, 32, 33]. Spyropoulus et al. [25] reported a much higher VTE recurrence risk of approximately 16% in their study. A likely explanation for this difference is the higher proportion of patients with malignancies and the variable duration of anticoagulation treatment and follow up. One meta-analysis of 11 NOAC trials showed that the risk of thromboembolism was higher in high body weight group compared with non-high body weight group among VTE patients (RR, 1.23; 95% CI 1.00–1.53; *p* = 0.05) which led to the belief that NOACs may be inappropriate in patients with higher body weight [34]. However, the control group in this study was the non-high body weight population and not high body weight patients on VKA, the conventional first-line oral anticoagulant in the morbidly obese. According to more recent reports and our findings, NOACs may be non-inferior or superior to VKAs when compared head-to-head [29–31]. A study of the Dresden NOAC Registry found that obese patients had lower rates of major bleeding compared with normal weight patients, which is consistent with the lower rates of major bleeding conferring superiority to xabans in the morbidly obese patients with VTE in our study [33].

In light of strong evidence from several meta-analyses and large retrospective cohort studies, xabans have emerged as a safe alternative to VKAs in morbidly obese patients. Our study consistently showed that xabans exhibited not only non-inferior efficacy but also lower odds of major bleeding when compared with VKAs to treat VTE in the morbidly obese. Despite fixed dose regimens, there is no clinical data to reflect decreased effectiveness of xabans based on the theoretical risk of reduced peak concentrations, low bioavailability, or shorter half-lives with increasing body weight. Our findings support the use of xabans in treating morbidly obese patients with VTE, consistent with the updated guidelines of ISTH which approve the use of standard-dose rivaroxaban and apixaban in patients with high BMI.

### Limitations

Our study contains several limitations. We only had access to published material with no individual patient-level data available. The number of studies included in our study is also relatively small, with most of them being retrospective cohort studies. Because the number of included studies was less than 10, the assessment of publication bias lacks power and may not be accurate. There were differences in the baseline demographics and comorbidities of the cohorts, which may have impacted the recurrence of VTE. Unaccounted confounders may also be present in the studies, as in patients on xabans being more socioeconomically privileged and potentially having higher medication compliance compared to those on VKA, a cheaper, older medication. Details of baseline characteristics and specific indications of anticoagulation were unable to be captured from several studies as outcomes in the morbidly obese were displayed only as subgroup analysis. The inclusion and exclusion criteria also differed according to studies, especially with some studies excluding patients with malignancies while others not specifying such restrictions. These inter-study differences, including but not limited to patient characteristics, indications of anticoagulation, type of xaban, and inclusion/exclusion criteria, likely resulted in low to moderate heterogeneity in integrated outcomes. Further prospective cohorts or randomized clinical trials are required to generate more accurate data on the effectiveness and safety profiles of xabans in treating morbidly obese patients with VTE. Pos-hoc analysis of patient-level data, specific to the morbidly obese population, from all the relevant randomized clinical trials may also be of great benefit.

## Supplementary Information

**Additional file 1.** All supplementary tables and figures.

## Data Availability

The datasets supporting the conclusions of this article are included within the article and its Additional file 1. All data used in this study have been retrieved from publicly available published papers, so approval from the institutional review board was not required.
