# Supplementary material for: Factor Xa inhibitors versus vitamin K antagonist in morbidly obese patients with venous thromboembolism: a systematic review and meta-analysis
Source: BMC Cardiovasc Disord. 2023 Feb 22;23:100. doi: 10.1186/s12872-023-03067-4 (PMC9945392; doi:10.1186/s12872-023-03067-4)

Supplementary Table 1. Assessment of risk of bias using the Newcastle-Ottawa scale

Trials	Selection				Comparability	Outcome			Overall Score
	Exposed	Non-exposed	Exposure	Outcome		Assessment	Follow Up	Adequacy	
**Kushnir et al.**	1	1	1	1	2	1	1	1	9
**Sa et al.**	1	1	1	0	1	1	1	0	6
**Spyropoulos et al.**	1	1	1	1	2	1	1	1	9
**Perales et al.**	1	1	1	1	2	1	1	1	9
**Cohen et al.**[a]	1	1	1	0	1	1	1	0	6
**Cohen et al.**[b]	1	1	1	1	2	1	1	1	9
**Costa et al.**	1	1	1	0	1	1	1	1	7
**Crouch et al.**	1	1	1	0	2	1	1	1	8

[a]Retrospective cohort study
[b]Post-hoc analysis

Supplementary Table 2. Grading of Recommendations, Assessment, Development and Evaluations (GRADE) criteria for outcomes

Outcome	Trials	Risk of Bias	Imprecision	Inconsistency	Indirectness	Publication Bias	Certainty
Recurrent venous thromboembolism	8	Moderate	Moderate	Moderate	Moderate	Low	⊕⊕⊕◯
Major bleeding	8	Moderate	Moderate	Moderate	Moderate	Low	⊕⊕⊕◯
Clinically relevant non-major bleeding	5	Moderate	Moderate	Low	Moderate	Low	⊕⊕⊕◯

Supplementary Table 3. PRISMA Checklist

Section and Topic	Item #	Checklist item	Location where item is reported
**TITLE**			
Title	1	Identify the report as a systematic review.	1
**ABSTRACT**			
Abstract	2	See the PRISMA 2020 for Abstracts checklist.	2
**INTRODUCTION**			
Rationale	3	Describe the rationale for the review in the context of existing knowledge.	3-4
Objectives	4	Provide an explicit statement of the objective(s) or question(s) the review addresses.	3-4
**METHODS**			
Eligibility criteria	5	Specify the inclusion and exclusion criteria for the review and how studies were grouped for the syntheses.	4-5
Information sources	6	Specify all databases, registers, websites, organisations, reference lists and other sources searched or consulted to identify studies. Specify the date when each source was last searched or consulted.	4-5
Search strategy	7	Present the full search strategies for all databases, registers and websites, including any filters and limits used.	4-5
Selection process	8	Specify the methods used to decide whether a study met the inclusion criteria of the review, including how many reviewers screened each record and each report retrieved, whether they worked independently, and if applicable, details of automation tools used in the process.	4-5
Data collection process	9	Specify the methods used to collect data from reports, including how many reviewers collected data from each report, whether they worked independently, any processes for obtaining or confirming data from study investigators, and if applicable, details of automation tools used in the process.	4-5
Data items	10a	List and define all outcomes for which data were sought. Specify whether all results that were compatible with each outcome domain in each study were sought (e.g. for all measures, time points, analyses), and if not, the methods used to decide which results to collect.	4-5
	10b	List and define all other variables for which data were sought (e.g. participant and intervention characteristics, funding sources). Describe any assumptions made about any missing or unclear information.	4-5
Study risk of bias assessment	11	Specify the methods used to assess risk of bias in the included studies, including details of the tool(s) used, how many reviewers assessed each study and whether they worked independently, and if applicable, details of automation tools used in the process.	5-6
Effect measures	12	Specify for each outcome the effect measure(s) (e.g. risk ratio, mean difference) used in the synthesis or presentation of results.	6
Synthesis methods	13a	Describe the processes used to decide which studies were eligible for each synthesis (e.g. tabulating the study intervention characteristics and comparing against the planned groups for each synthesis (item #5)).	6
	13b	Describe any methods required to prepare the data for presentation or synthesis, such as handling of missing summary statistics, or data conversions.	6
	13c	Describe any methods used to tabulate or visually display results of individual studies and syntheses.	6
	13d	Describe any methods used to synthesize results and provide a rationale for the choice(s). If meta-analysis was performed, describe the model(s), method(s) to identify the presence and extent of statistical heterogeneity, and software package(s) used.	6
	13e	Describe any methods used to explore possible causes of heterogeneity among study results (e.g. subgroup analysis, meta-regression).	6
	13f	Describe any sensitivity analyses conducted to assess robustness of the synthesized results.	6
Reporting bias assessment	14	Describe any methods used to assess risk of bias due to missing results in a synthesis (arising from reporting biases).	5-6
Certainty assessment	15	Describe any methods used to assess certainty (or confidence) in the body of evidence for an outcome.	5-6
**RESULTS**			

Section and Topic	Item #	Checklist item	Location where item is reported
Study selection	16a	Describe the results of the search and selection process, from the number of records identified in the search to the number of studies included in the review, ideally using a flow diagram.	Figure 1
	16b	Cite studies that might appear to meet the inclusion criteria, but which were excluded, and explain why they were excluded.	Figure 1
Study characteristics	17	Cite each included study and present its characteristics.	6, Table 1
Risk of bias in studies	18	Present assessments of risk of bias for each included study.	7, Table S1-S2
Results of individual studies	19	For all outcomes, present, for each study: (a) summary statistics for each group (where appropriate) and (b) an effect estimate and its precision (e.g. confidence/credible interval), ideally using structured tables or plots.	Figure 2-4
Results of syntheses	20a	For each synthesis, briefly summarise the characteristics and risk of bias among contributing studies.	6
	20b	Present results of all statistical syntheses conducted. If meta-analysis was done, present for each the summary estimate and its precision (e.g. confidence/credible interval) and measures of statistical heterogeneity. If comparing groups, describe the direction of the effect.	7-8, Figure 2-4
	20c	Present results of all investigations of possible causes of heterogeneity among study results.	7-8, Figure 2-4
	20d	Present results of all sensitivity analyses conducted to assess the robustness of the synthesized results.	7-8, Figure S2-S3
Reporting biases	21	Present assessments of risk of bias due to missing results (arising from reporting biases) for each synthesis assessed.	7, Table S1-S2
Certainty of evidence	22	Present assessments of certainty (or confidence) in the body of evidence for each outcome assessed.	7, Table S2
**DISCUSSION**			
Discussion	23a	Provide a general interpretation of the results in the context of other evidence.	8-9
	23b	Discuss any limitations of the evidence included in the review.	10-11
	23c	Discuss any limitations of the review processes used.	10-11
	23d	Discuss implications of the results for practice, policy, and future research.	9-10
**OTHER INFORMATION**			
Registration and protocol	24a	Provide registration information for the review, including register name and registration number, or state that the review was not registered.	5
	24b	Indicate where the review protocol can be accessed, or state that a protocol was not prepared.	5
	24c	Describe and explain any amendments to information provided at registration or in the protocol.	5
Support	25	Describe sources of financial or non-financial support for the review, and the role of the funders or sponsors in the review.	14
Competing interests	26	Declare any competing interests of review authors.	14
Availability of data, code and other materials	27	Report which of the following are publicly available and where they can be found: template data collection forms; data extracted from included studies; data used for all analyses; analytic code; any other materials used in the review.	4, 14

From: Page MJ, McKenzie JE, Bossuyt PM, Boutron I, Hoffmann TC, Mulrow CD, et al. The PRISMA 2020 statement: an updated guideline for reporting systematic reviews. BMJ 2021;372:n71. doi: 10.1136/bmj.n71

Supplementary Table 4. Inclusion and Exclusion Criteria of Selected Studies

Author	Inclusion Criteria	Exclusion Criteria
Kushnir et al.	BMI ≥40kg/m^2<br>Anticoagulation for either VTE or AF	Patients with both VTE and AF<br>Anticoagulation indication other than VTE and AF<br>Non-compliance<br>No follow up after anticoagulation
Sa et al.	BMI ≥30kg/m^2 or body weight ≥120kg<br>Objectively confirmed acute VTE	Anticoagulation for conditions other than VTE<br>Cancer-related thrombosis<br>Missing data
Spyropoulous et al.	Diagnosis of morbid obesity<br>Anticoagulation with either rivaroxaban or warfarin within 28 days of VTE diagnosis	Diagnosis of VTE or AF prior to date of diagnosis<br>Previously on anticoagulation
Perales et al.	BMI ≥40kg/m^2 or body weight ≥120kg<br>Anticoagulation with either rivaroxaban or warfarin for AF or VTE during admission	Previously on anticoagulation<br>Bioprosthetic or mechanical heart valve<br>Hemodialysis
Cohen et al.[a]	BMI ≥30kg/m^2 and BMI ≥40kg/m^2<br>Age ≥18 years<br>Diagnosis of VTE<br>Anticoagulation with warfarin or apixaban	AF or mechanical heart valve<br>Prior VTE diagnosis within past 6 months<br>Active cancer within past 6 months or 30 days after<br>Inferior vena cava filter<br>Antiphospholipid syndrome<br>Pregnancy
Cohen et al.[b]	BMI ≥40kg/m^2 or body weight ≥120kg<br>Age ≥18 years<br>Diagnosis of VTE<br>Anticoagulation with subcutaneous enoxaparin and warfarin or apixaban	Active bleeding<br>High risk of bleeding<br>Other contraindication to anticoagulation<br>Cancer-related VTE<br>Provoked VTE in absence of persistent risk factor for recurrence<br>Less than 6 months of anticoagulation<br>Other indication for anticoagulation, dual antiplatelet therapy, or aspirin ≥165mg per day<br>Treatment with cytochrome P450 3A4 inhibitors
Costa et al.	BMI ≥30kg/m^2 and BMI ≥40kg/m^2<br>Diagnosis of VTE<br>Anticoagulation with warfarin or rivaroxaban within 7 days of VTE diagnosis	Evidence of cancer<br>Anticoagulation for conditions other than VTE<br>Prior oral anticoagulation use
Crouch et al.	BMI ≥40kg/m^2 or actual body weight ≥120kg	Mechanical heart valve

	Age >18 years<br>Continuation of anticoagulant at discharge	Severe liver disease<br>Pregnancy<br>Taking a medication contraindicated for concomitant use with DOAC or warfarin therapy

[a]Retrospective cohort study
[b]Post-hoc analysis
Abbreviations: AF = atrial fibrillation; BMI = body mass index; VTE = venous thromboembolism

Supplementary Figure 1. Funnel plot of recurrent venous thromboembolism reported in the selected studies

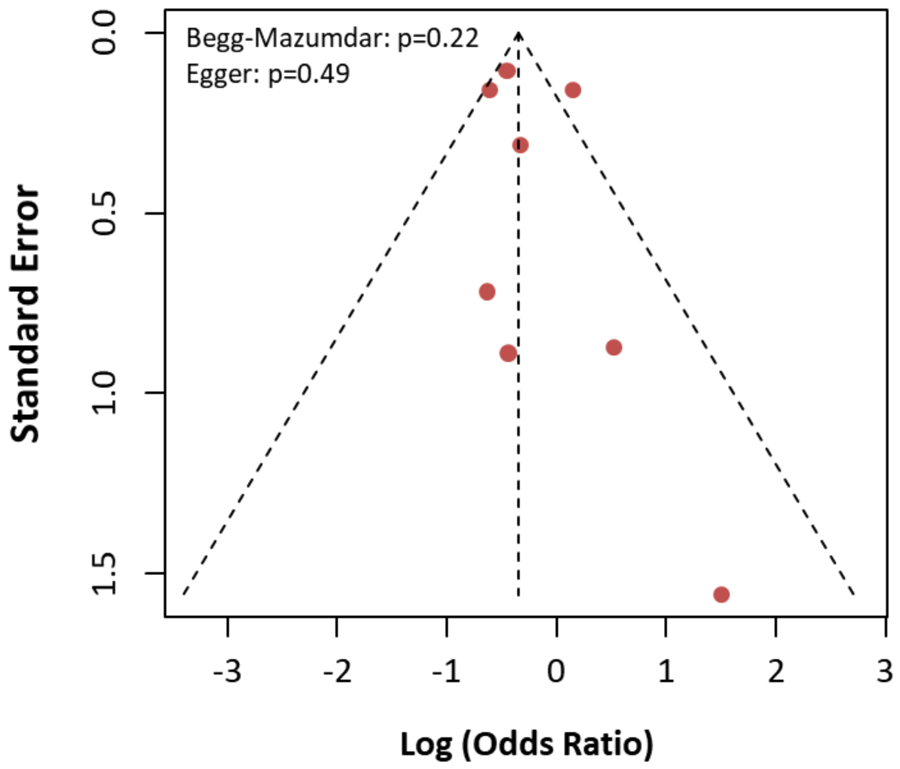

Supplementary Figure 2. Sensitivity analysis excluding studies with high risk of bias

Study	Xabans Events	Xabans Total	VKA Events	VKA Total	Weight	Odds Ratio MH, Random, 95% CI
Kushnir et al.	4	199	2	167	5.4%	1.69 [0.31; 9.36]
Spyropoulos et al.	87	2890	75	2890	30.5%	1.16 [0.85; 1.59]
Perales et al.	2	47	4	62	5.2%	0.64 [0.11; 3.68]
Cohen et al.	3	126	6	137	7.4%	0.53 [0.13; 2.18]
Costa et al.	66	1697	118	1697	30.6%	0.54 [0.40; 0.74]
Crouch et al.	14	314	48	785	21.0%	0.72 [0.39; 1.32]
**Total (95% CI)**		**5273**		**5738**	**100.0%**	**0.78 [0.51; 1.19]**

Heterogeneity: $Tau^2 = 0.1318$; $Chi^2 = 12.85$, df = 5 (P = 0.02); $I^2 = 61\%$

Test for overall effect: Z = -1.15 (P = 0.25)

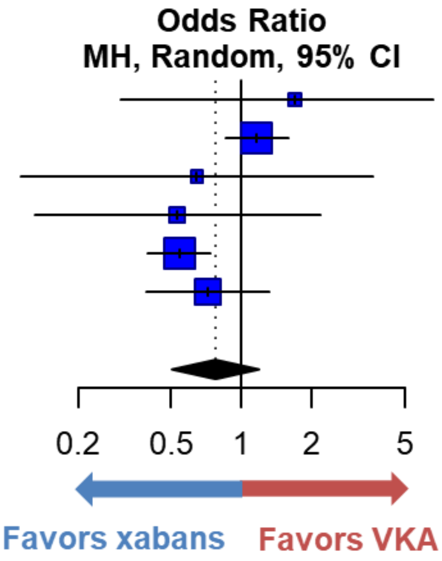

Supplementary Figure 3. Sensitivity analysis excluding trials that used body weight ≥120kg to define morbid obesity

Study	Xabans Events	Xabans Total	VKA Events	VKA Total	Weight	Odds Ratio MH, Random, 95% CI
Kushnir et al.	4	199	2	167	4.2%	1.69 [0.31; 9.36]
Spyropoulos et al.	87	2890	75	2890	28.7%	1.16 [0.85; 1.59]
Cohen et al.	130	7411	337	12340	32.5%	0.64 [0.52; 0.78]
Cohen et al.	3	126	6	137	5.9%	0.53 [0.13; 2.18]
Costa et al.	66	1697	118	1697	28.8%	0.54 [0.40; 0.74]
**Total (95% CI)**		**12323**		**17231**	**100.0%**	**0.74 [0.51; 1.08]**

Heterogeneity: $Tau^2 = 0.1001$; $Chi^2 = 14.82$, df = 4 ($P < 0.01$); $I^2 = 73\%$
Test for overall effect: $Z = -1.55$ ($P = 0.12$)

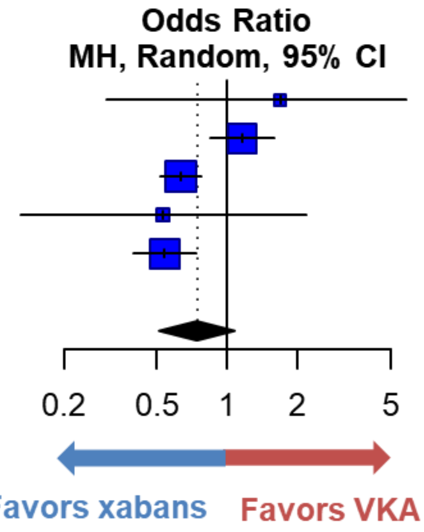

Supplement: Supplementary file 1 — Additional file 1. All supplementary tables and figures. [file 12872_2023_3067_MOESM1_ESM.pdf]
